# BAK multimerization for apoptosis, but not bid binding, is inhibited by negatively charged residue in the BAK hydrophobic groove

**DOI:** 10.1186/1476-4598-12-65

**Published:** 2013-06-19

**Authors:** Abul Azad, Alan Storey

**Affiliations:** 1 Department of Oncology, Weatherall Institute of Molecular Medicine, University of Oxford, Oxford OX3 9DS, UK

**Keywords:** Apoptosis, Mitochondria, BCL-2, BAK, BH3, Cytochrome *c*, Tyrosine Phosphorylation, DNA damage

## Abstract

**Background:**

BCL-2 family proteins BAK and BAX orchestrate outer mitochondrial membrane permeabilization (MOMP) during apoptosis by forming pores in the membrane to release apoptogenic factors that commits a cell to death. BAK and BAX therefore function as a ‘point of no return’ in the apoptotic cascade. BAK activation is a multi-step process involving conformational changes, mediated by BH3-only proteins or p53, which lead eventually to oligomerization and pore formation. Further, recent reports show that BAK activation is also linked to and dependent upon dephosphorylation of both tyrosine and serine residues.

**Findings:**

We hypothesized that phosphorylation of BAK at tyrosine residue 110 (Y110) was functionally important during the BAK activation process. BAK/BAX double knockout HCT116 cells expressing a phosphor-mimetic BAK mutant (BAK Y110E), showed impaired dimerization and multimerization capacity when treated with either UV irradiation or etoposide when compared to cells reconstituted to express wild-type BAK. The Y110E mutant also showed decreased release of cytochrome *c* from isolated mitochondria challenged with tBid protein, resulting in a failure to activate caspase 3. Interestingly, co-immunoprecipitation experiments suggest that a negative charge at this residue may be important for the recruitment of Bid to BAK, but conversely that this also impairs BAK:BAK interactions.

**Conclusion:**

These findings implicate dephosphorylation of Y110 as having an important mechanistic role in BAK activation, and underscores how post-translational modifications are intimately linked and coupled to the protein-protein interactions required for BAK activation during apoptosis.

## Findings

The abrogation of apoptotic responses is a common feature of almost all cancer cells. Whether or not a cell undergoes apoptosis in response to stress depends on responses following the receipt of signals generated by cellular damage. The mitochondrial apoptotic pathway is regulated by BCL-2 proteins that can be divided into different pro- and anti-apoptotic groups depending on their structure and function [1]. Pro-apoptotic BH3-only proteins, such as Bid, Bim and Noxa, are required for the activation of multi-domain BCL-2 effector proteins BAK and BAX. BAK and BAX constitute a pre-requisite gateway for mitochondrial apoptosis to proceed, indeed cells lacking both BAK and BAX fail to undergo mitochondrial apoptosis [2]. The BAK responds to a myriad of death signals and plays a key role in executing mitochondrial apoptosis, effecting MOMP through oligomerization of the protein that releases apoptogenic factors including cytochrome *c*, that in turn lead to downstream activation of caspase 3. BAK activation is complex, involving a number of intra-molecular conformational changes leading to dimerization followed by oligomerization [2-4]. An early event during BAK activation is a conformational change towards to N-terminus of the protein [5]. This is followed by exposure of the BAK BH3 domain that then inserts into a hydrophobic groove on another BAK molecule leading to dimer formation [6]. Resultant homodimers then can form higher order structures via interaction between α6:α6 helices [7]. BAK N-terminal conformational change can be brought about by one of two mechanisms: first, by the transient binding of BH3-only proteins (such as tBid) [8] to the BAK hydrophobic groove [2,9], or alternatively by the binding of p53 to residues near the BAK N-terminus [10-12]. Binding of BH3 proteins such as tBid to the BAK hydrophobic groove occurs with high affinity, but is necessarily transient as this same region of BAK is also required to nucleate BAK multimerization. Recently a model of BAK activation that tries to take into account the differing affinities of BH3 proteins for both pro- and anti-apoptotic BCL-2 proteins has been proposed [13].

In healthy, otherwise undamaged cells, BAK is present almost exclusively in a very heavily phosphorylated form. We recently demonstrated that BAK activation for apoptosis induction is closely linked to, and indeed dependent upon, specific dephosphorylation events on the protein [14]. The initial event in the BAK activation process is dephosphorylation at tyrosine 108 (Y108), an obligatory step that is required to permit conformational change by BH3 or p53 proteins [14,15]. Further, we found that a subsequent PP2A-mediated dephosphorylation of BAK at serine 117 (S117) was required both for BH3 proteins to gain access to the BAK hydrophobic groove, and permit BAK dimerization via BAK-BH3:BAK-groove interactions [16].

**Figure 1:**
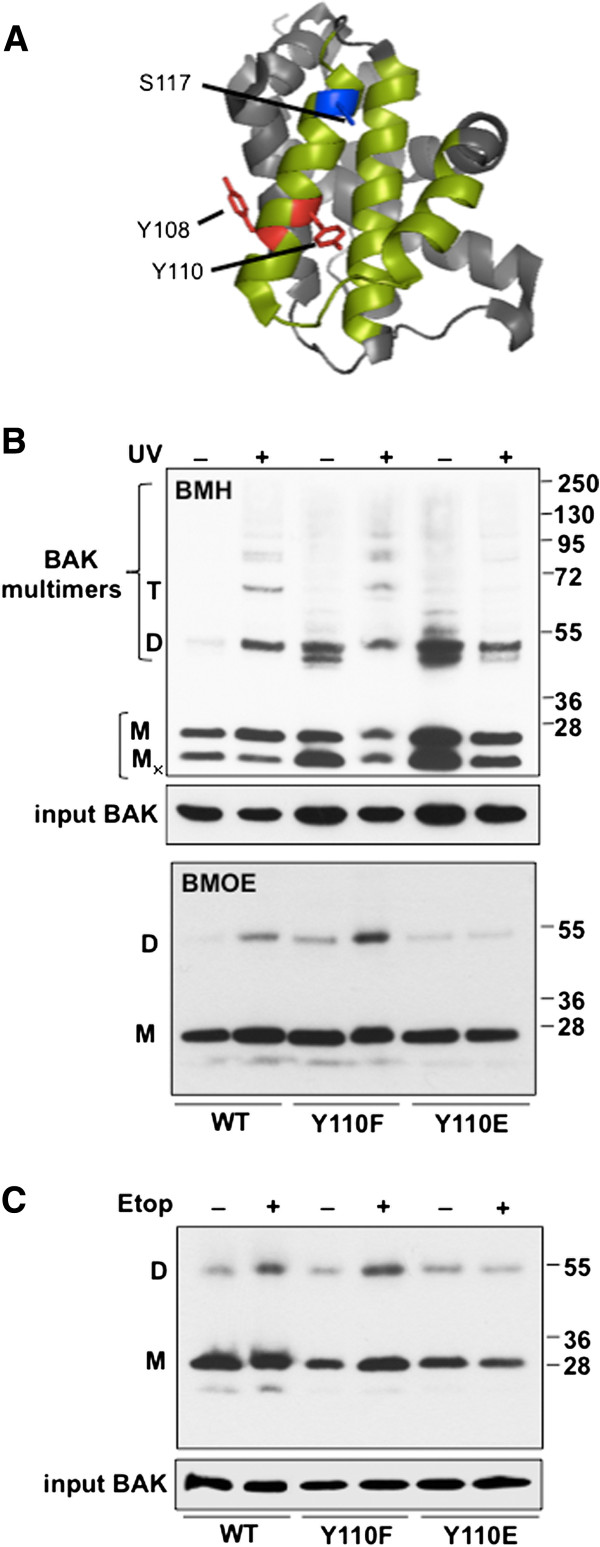
**Effects of mutation of tyrosine 110 (Y110) on the ability of BAK to form multimers following DNA damage.** (**A**) PyMol generated model of BAK structure using PBD file 2IMS. Locations on the α4 helix of Y108 (red), together with S117 (blue) and Y110 (red) where the side chains of these residues face the hydrophobic surface groove (green), are indicated. Both the BH3 domain and BH1 encompassing α2-α5 helices (residue 70–145) constitutes the hydrophobic surface groove. (**B**) BAK multimerization following DNA damage was analysed in HCT116-BAK cells as described in [16]. HCT116 DKO cells were reconstituted with the WT BAK, Y110F or Y110E BAK proteins. Mitochondria were isolated from cells expressing WT, Y110F and Y110E in ±UV 10mJ/cm^2^ for 8 hrs. 100 μg of mitochondria were cross-linked with either BMH (top panel) or BMOE (bottom panel). BAK was detected by western blot using a rabbit anti BAK monoclonal (abcam, Y164). The input was the 5% of mitochondrial extract used in the cross-linking studies to ensure equal loading (middle panel). Non cross-linked BAK runs as a monomer (M) and also as an intra-molecularly linked monomer (Mx). BAK dimers (D) and trimers (T) and higher order structures are indicated. Image is representative of 3 independent experiments. (**C**) Similar multimerization experiment to (B) was performed with HCT116 cells expressing WT BAK, Y110F or Y110E proteins ± 50 μM etoposide treatment for 8 hrs. Mitochondrial proteins were cross-linked with BMOE as described previously and detected as above. Note that BMOE generates only dimer forms of BAK.

During our investigations into the role of phosphorylation in regulating BAK activation, we reported in mass spectrometry analysis that BAK was also phosphorylated at residue Y110 [14]. Mutation of this residue to mimic either the dephosphorylated or phosphorylated forms of the protein (BAK mutants Y110F and Y110E respectively) did not affect the ability of BAK to undergo N-terminal conformational change [14]. However, modelling using PyMol based on BAK structure 2IMS, suggested that phosphorylation at Y110 might impinge upon the ability of BH3 proteins to bind BAK and may also inhibit BAK multimerization, as the Y110 side chain, like that of S117, may occlude access to the hydrophobic groove (Figure 1A). To test this idea, we performed multimerization assays using sub-cellular fractions enriched in mitochondria from HCT116^*bak*−/−*bax*−/−^ cells that had been reconstituted to express either wild-type (WT) or mutant BAK proteins (HCT-BAK cells). Cross-linking reactions were performed with the sulfhydryl-to-sulfhydryl crosslinker BMH (1, 6-bismaleimidohexane, Pierce) on sub-cellular fractions enriched in mitochondria, then analysed by western blotting to detected BAK multimers, as previously described [14,16]. Following DNA damage by UV, BAK dimers, trimers and higher-order complexes were observed with WT BAK and the Y110F mutant, but multimer formation was impaired severely by the Y110E mutation (Figure 1B, upper panel). As we have noted previously, BAK cross-linking with BMH can be problematic with the formation of a faster migrating intra-molecularly cross-linked monomeric BAK protein (M_x_) and generation of non-specific dimer bands without any DNA damage. A further cross-linking experiment a different sulfhydryl crosslinker, BMOE (Bismaleimidoethane), that generates only BAK dimers and at the same time minimizes the detection of both intra-molecularly cross-linked BAK monomers and non-specific dimer forms [16], supported the previous experiments with dimers being detected only when WT or the Y110F mutant was used (Figure 1B, lower panel). When the experiment was repeated using etoposide as the apoptotic inducer in place of UV, again dimers were readily detected in cells expressing the WT and Y110F proteins, but were not generated by the Y110E mutant (Figure 1C). The stronger dimer band present generated by the Y110F mutant compared to WT we suppose may be due to the mutant forming dimers more readily if the WT protein was not fully dephosphorylated following UV damage.

**Figure 2:**
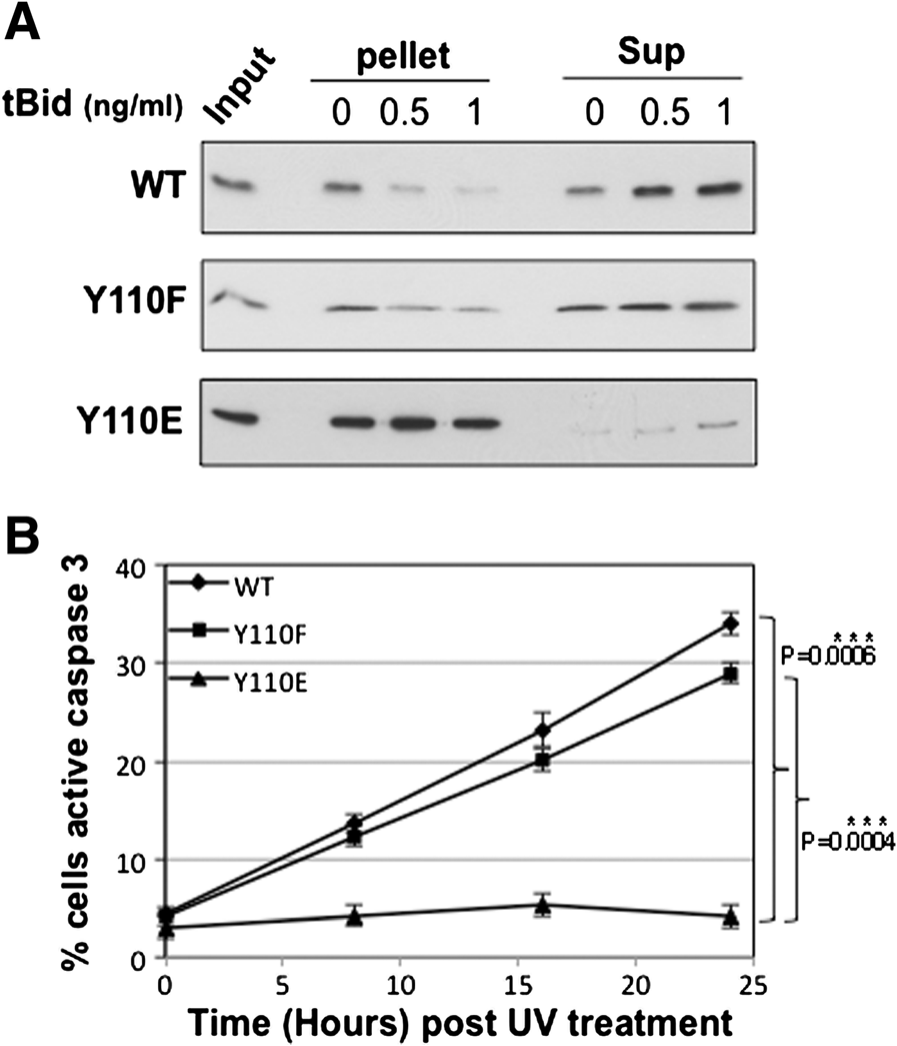
**Cytochrome *c *release and caspase3 activation in BAK mutant cells.** (**A**) Cytochrome *c* release assays were performed essentially as described [15,16]. Mitochondria were isolated from HCT116-WT BAK (top panel), HCT1116-Y110F (middle panel) and HCT116-Y110E (bottom panel) cells and incubated with recombinant tBid (1 ng/μl, R&D). After incubation at 37°C for 30 min, mitochondria were separated into pellet and supernatant fractions by centrifugation. Cytochrome *c* levels were analysed by western blotting with anti-cytochrome *c* antibody (BD Pharmingen) in both pellet (indicating retention in mitochondria) and supernatant (sup; released from mitochondria) fractions. In all cytochrome *c* release assays, an equal amount of isolated mitochondria not treated with tBid was used as input. (**B**) Activation of caspase 3 was analysed by FACS using an antibody that specifically recognises only cleaved (therefore active) form of caspase 3 in HCT116 cells expressing WT BAK, BAK-Y110F or BAK-Y110E mutants treated with ± 10mJ/cm^2^ UV. Data are the mean percentage of 3 independent experiments, ±S.E.M.

The failure of the BAK Y110E mutant to dimerize or multimerize we reasoned may be due to interference with the binding of BH3 proteins, as observed previously for the S117E phosphor-mimic [16]. We therefore tested the ability of the BH3-activator protein tBid to release cytochrome *c* from mitochondrial preparations using a previously established method [16,17]. When purified tBid protein was incubated with mitochondrial preparations from cells expressing either the WT or Y110F BAK proteins, cytochrome *c* was readily released into the supernatant (Figure 2A). Consistent with the multimerization assays, tBid was unable to release cytochrome *c* from mitochondrial preparations from the BAK-Y110E mutant cells compared to the HCT116-BAK or HCT116-BAKY110F cells (Figure 2A). The small quantity of cytochrome *c* that was detected in the supernatant fraction derived from the Y110E mutant might be due to the mutant being very inefficient at releasing cytochrome *c* since the substituted amino acid may not correctly mimic a phosphor-tyrosine residue, however we have noted that processing of the samples leads to a degree of leakiness of the mitochondrial preparations where mitochondria expressing BAK mutants we find to be more fragile compared to cells expressing WT BAK, as previously noted [16]. Likewise, the Y110F mutant showed amounts of cytochrome *c* retained in the pellet fraction decreased somewhat when mitochondrial preparations were treated with increasing amounts of tBid, but by comparison cytochrome *c* was readily detected in the supernatant fraction even in the absence of tBid, and did not increase to the same extent as observed for the WT BAK protein. To investigate further whether the cytochrome *c* release was likely to be functionally significant and bypass potential problems of mitochondrial fragility, we performed caspase 3 activation assays using a FITC-conjugated antibody that recognizes only activated caspase 3 using FACS on intact cells. This revealed that UV irradiation resulted in an increase in caspase 3 activation in BAK WT or Y110F cells, but in marked contrast no caspase 3 activation was detected in UV-irradiated Y110E cells, findings in-line with and supporting the cytochrome *c* release experiment (Figure 2B). We conclude that the Y110E mutation interferes with the ability of BAK to form dimers and multimers, and that this results in the failure to release cytochrome *c* and activate caspase 3.

**Figure 3:**
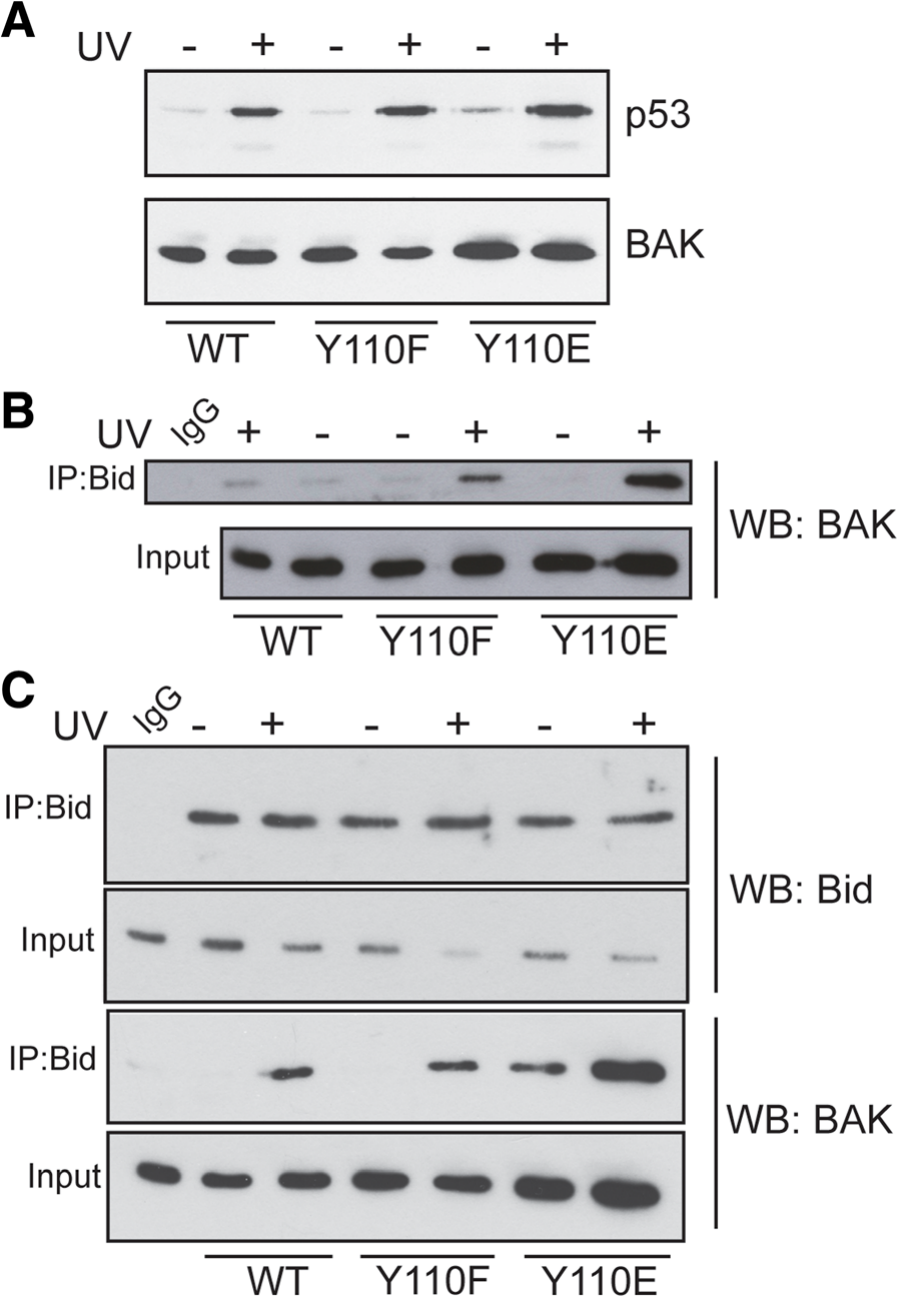
**Effects of BAK mutations on p53 mitochondrial translocation and ability to bind Bid.** (**A**) Translocation of p53 into mitochondria after DNA damage. Mitochondria were isolated from cells expressing WT BAK, BAK-Y110F or BAK-Y110E mutant proteins following treatment with ± 10mJ/cm^2^ UV. p53 was detected by western blot using the DO1 antibody (upper panel). Expression levels of the BAK proteins (detected as above) are also shown (lower panel). (**B**) Total cell extracts were prepared from HCT116 cells expressing WT BAK, Y110F or Y110E mutants following treatment with ± 10mJ/cm^2^ UV with extraction buffer (20 mM Tris–HCl pH7.4, 135 mM NaCl, 1.5 mM MgCl2, 1 mM EGTA, 10% glycerol, protease and phosphatase inhibitor cocktail containing 1% CHAPS). Immunoprecipitations were performed with anti Bid antibody (Cell Signalling) and BAK was detected by immunoblotting with rabbit anti-BAK (Y164, abcam). The input was 5% of the extracts used in immunoprecipitation reactions. Here and in panel (C) below, the non-UV treated WT cell extract was used for the IgG control immunoprecipitation. (**C**) Mitochondrial-enriched sub-cellular fractions were prepared from cells expressing WT or BAK mutants ± UV treatment as outlined in Figure 1. Immunoprecipitations for Bid and detection of BAK by western blotting were as in (B) above. The input was 5% of the extracts used in immunoprecipitation reactions.

In previous studies, we found that neither the BAK Y110F nor Y110E mutations had any effect on BAK being able to undergo the N-terminal conformational change associated with an early step in BAK activation, indicating that the mutations did not perturb the overall BAK structure [14]. Since either p53 or BH3 proteins bring about BAK conformational change by different routes [16], we next asked whether the BAK mutants recruited p53 to the mitochondria as efficiently as the WT protein. The p53 binding site on BAK is located near the N-terminus of the protein involving residues E24/E25 [12,16], and p53 recruitment to the mitochondria is dependent on BAK expression [10,18,19]. Following DNA damage by UV irradiation, we found that p53 was readily recruited to the mitochondria as was detected by western blot in mitochondrial extracts of cells expressing the WT or either of the two mutant BAK Y110F or Y110E (Figure 3A). As the Bid BH3 sequence interacts with the BAK hydrophobic groove, we next examined whether Bid was able to Co-IP BAK following UV irradiation. As expected, Bid was able to pull down BAK protein following UV irradiation from whole cell extracts of cells expressing either the WT or Y110F mutant BAK proteins (Figure 3B). Somewhat surprisingly, Bid was able to pull down more BAK from the Y110E cells when compared to cells expressing either WT or the Y110F mutant (Figure 3B). To explore further the role of a negative charge at Y110 on the recruitment of Bid to BAK, we performed similar IP/western blot experiments using mitochondrial enriched preparations. We found that similar levels of Bid were able to be immunoprecipitated in the three BAK cell lines expressing either WT, Y110F or Y110E mutants, irrespective of whether the cells has been treated with UV or not (Figure 3C). As before, WT or Y110F BAK proteins were only co-precipitated with Bid following UV damage, and that a greater amount of the Y110E BAK mutant we again found was readily pulled down by Co-IP with Bid following UV treatment (Figure 3C). However, in these mitochondrial enriched subcellular fractions we were also now able to detect BAK that had been pulled down by the Bid antibody even in the absence of UV treatment. Together these findings provides the first evidence that a negative charge located in the BAK hydrophobic surface groove may be a factor important in the recruitment of BH3 proteins such as Bid to BAK.

Blockade of the BAK hydrophobic groove by phosphorylation of S117, or an S117E mutant, each impair binding of either BH3 peptides or proteins to BAK. Located further along the same helix as S117 but also facing the hydrophobic BAK surface pocket, we now identify a role for Y110 in the BAK activation process. Taken together, our results identify for the first time a novel and important bi-functional role for post-translational modifications at Y110. Binding of BH3 proteins to BAK has been reported to occur with high affinity yet the interaction must be transient [20], as the same hydrophobic surface interface is required to nucleate BAK dimerization. We recently reported that phosphorylation of BAK at S117 blocks access of BH3 sequences of Bid and BAK to the hydrophobic pocket on BAK. In contrast, these findings suggest that while the Y110E mutant interferes with the ability to form stable BAK-BH3:BAK-groove interactions for dimerization, a negative charge at this site may also be a positive factor that is involved in the recruitment of BH3-containing proteins, such as Bid, to BAK. This implies that dephosphorylation of S117 must occur prior to dephosphorylation of Y110 in order to permit Bid recruitment, however the exact timing of these events and whether binding of other BH3-containing BCL-2 family proteins is also affected by this modification remains to be established.

Findings presented here further underscore the importance of post-translational modifications in regulating different steps in BAK activation, and may reveal new avenues to either potentiate or inhibit BAK activity through modulation of the phosphorylation status of the protein.

## Competing interests

The authors state that they have no competing interests.

## Authors’ contributions

AA and AS designed the experiments; AA performed all experimental procedures; AA and AS analysed data and generated images, and prepared and approved the final version of the manuscript.
